# Dr. Cornelius H. Bartlett

**Published:** 1909-02

**Authors:** 


DR. CORNELIUS H. BARTLETT, of Olean, N. Y., died at his home in that city January 22, 1909, aged 83 years. He was one of the best known physicians in the "Southern Tier," having practised his profession in that region for nearly fifty years. He was a member of the Medical Societies of the county and state, and of the American Medical Association. He graduated at Geneva Medical College in 1849. Mrs. Bartlett who was ill with pneumonia at the time of Dr. Bartlett's death survived her husband but two days. A double funeral was held January 26, and both buried in Mount View Cemetery at Olean.

